# Capromorelin oral solution (ENTYCE®) increases food consumption and body weight when administered for 4 consecutive days to healthy adult Beagle dogs in a randomized, masked, placebo controlled study

**DOI:** 10.1186/s12917-016-0925-z

**Published:** 2017-01-05

**Authors:** Bill Zollers, Linda Rhodes, Ernst Heinen

**Affiliations:** 1 Norbrook Inc., Lenexa, KS USA; 2 Independent Consultant, Durham, NH USA; 3 Aratana Therapeutics, Inc., 11400 Tomahawk Creek Parkway, Suite 340, Leawood, KS USA

**Keywords:** Ghrelin receptor agonist, Growth hormone secretagogue, Anorexia, Appetite stimulation

## Abstract

**Background:**

Dogs can suffer from inappetence caused by a variety of medical conditions. This may present as anorexia (complete loss of appetite), hyporexia (decreased appetite) or dysrexia (change in food preferences). A drug with a new mechanism of action, capromorelin, has potential to stimulate appetite in dogs. Capromorelin is a ghrelin receptor agonist, which mimics the action of endogenous ghrelin. It is a member of the growth hormone secretagogue (GHS) class of drugs. Capromorelin oral solution (ENTYCE®) was tested in healthy adult male and female Beagle dogs (*n* = 6 males and 6 females per group) for its effect on food consumption and body weight. A randomized, masked, placebo controlled study was conducted to measure the effects of a daily 3 mg/kg oral dose given over 4 days. Dogs were observed for clinical signs, physical examinations were completed prior to and at the end of treatment, and blood was drawn before and after treatment for evaluation of serum chemistry and hematology parameters.

**Results:**

Capromorelin was well-tolerated, with no abnormalities seen on physical examination or clinical pathology. Some dogs showed increased salivation. Capromorelin treated dogs had increased mean (±SD) food consumption compared to placebo treated dogs (60.55 ± 39.87% versus -11.15 ± 14.23% respectively, *P* < 0.001). Treated dogs also had increased mean body weights compared to placebo treated dogs (5.96 ± 1.76% versus 0.053 ± 1.14% respectively, *P* < 0.001).

**Conclusions:**

This study supports the effectiveness of capromorelin oral solution as an appetite stimulant in dogs. Treatment with the oral solution resulted in dramatic increases in appetite, as measured by food consumption, of over 60% compared to placebo. The drug was well tolerated. Capromorelin is the first ghrelin receptor agonist developed for appetite stimulation in any species, and represents a novel mechanism of action for this clinical use.

## Background

Dogs can present at the veterinary clinic with anorexia (a complete lack of eating), hyporexia (a decrease in food intake) or dysrexia (altered preferences or patterns of eating). Reduced appetite (inappetence) is a common, nonspecific clinical sign in dogs that is associated with many diseases. Owners may notice a reduced or changed appetite as the first indication that something may be wrong with their dog’s health. Reduced food intake contributes to weight and muscle loss and can cause significant nutritional deficiencies, which can further exacerbate many medical conditions and may even have direct negative effects [1, 2]. Owners can become distressed when their dog does not eat normally. Reduced appetite is one of the most common contributing factors to an owner’s decision to euthanize their dog [3]. Therefore, stimulating appetite would aid in providing optimal nutrition for the dog which is critical for managing many disease conditions, and would be a positive sign for the veterinarian and pet owner. Restoring optimal nutrition could help dogs recover from surgery or injury faster by increasing strength, immune function and wound healing. In addition, increased appetite could possibly shorten a hospital stay or even be a benefit by providing time so that the dog’s underlying issue can be identified and addressed [1, 4]. Restoring appetite is an important component of many treatment plans for sick dogs.

Discovery of a drug that may be helpful in treating dogs with reduced appetite began with the creation of a new class of drugs, the growth hormone secretagogues (GHS) - a class of small molecule compounds discovered in the mid-1990s that stimulate the release of growth hormone (GH) and may be useful in treatment of anorexia and cachexia [5]. It was subsequently discovered that GHS compounds mimic ghrelin, the hormone that is secreted from endocrine cells in the stomach and stimulates appetite and food intake in humans [6].

Capromorelin is an orally active potent growth hormone secretagogue receptor (GHS-R) agonist that mimics the action of ghrelin and acts directly on the hunger centers of the hypothalamus to stimulate appetite and enhance food consumption [5]. Since there were no FDA approved veterinary drugs for inappetence, veterinarians currently uses unapproved alternatives that were not developed for the purpose of appetite stimulation. Examples of unapproved drugs used to stimulate appetite in dogs and cats include mirtazapine, cyproheptadine, maropitant, benzodiazepines and glucocorticoids. The problem with many of these unapproved alternatives is that safe and efficacious doses have not been established for pets. The FDA has approved capromorelin (ENTYCE®) and this approval represents the first regulatory approval, either veterinary or human, of a drug with this mechanism of action, i.e. a ghrelin receptor agonist.

Capromorelin oral solution has been shown to increase food consumption, body weight and GH and insulin-like growth factor 1 (IGF-1) secretion in a small study in laboratory Beagle dogs [7]. A 12-month oral safety study in adult dogs has shown that long-term daily oral administration of capromorelin at a dose approximately 17.5 times that of the dose tested in the present study is safe [8].

Client–owned dogs that are experiencing reduced appetite due to a variety of clinical conditions may not be as sensitive to the effects of a ghrelin receptor agonist such as capromorelin. To confirm that capromorelin could prove efficacious in dogs with clinical signs of inappetence, two masked, placebo-controlled, randomized, multi-site clinical studies in client-owned dogs have been conducted in veterinary clinics in the USA. First, a pilot study was conducted in 36 dogs presenting with reduced appetite from a variety of clinical conditions. Seven days of treatment with capromorelin was associated with increased body weight, and owner’s assessed their dogs appetites were increased when compared to placebo treated dogs [9]. In a recent clinical study in 244 dogs at over 30 veterinary clinics, dogs experiencing inappetence from a variety of conditions were treated with 3 mg/kg capromorelin for 4 days. This study demonstrated a statistically significant improvement in appetite as measured by an owner appetite assessment (*P* = 0.0078) and an increase in body weight (*P* = 0.0004) in the capromorelin treated group compared to the placebo group [10].

In these clinical studies, although owners assessed their dogs as showing an increased appetite, and dogs gained weight, there was no direct evidence of increased food intake, due the difficulties of measuring food intake in a pet at home who may be fed a variety of foods by various household members, and food intake data may also be confounded due to multiple pets in the household.

The purpose of this laboratory study was to confirm in a well controlled environment, where food intake could be accurately measured, that a 3 mg/kg daily treatment of capromorelin oral solution given for 4 days to male and female laboratory dogs would increase food consumption and body weight.

## Methods

### Animals and housing

Twenty-four, non-naïve healthy Beagle dogs, bred for research purposes (Marshall BioResources, North Rose, NY), were divided into 2 treatment groups (*n* = 6 males and 6 females/group). Dogs ranged from 13.3 to 13.8 months of age at the study start. Body weights ranged from 6.5 to 9.4 kg for females and 10.0 to 12.6 kg for males at randomization. Dogs were housed individually in stainless steel cages with controlled temperature (18 to 29 °C), relative humidity ranging from 30 to 70% and a photoperiod of 12 h of light alternating with 12 h of darkness. All dogs were under the care of a licensed veterinarian. Water was provided *ad libitum* (see below for information about feeding). At the end of the study (following weighing on Day 4), all dogs were returned to the facility colony.

### Treatment

The study tested capromorelin flavored oral solution (ENTYCE®) with 30 mg/ml of capromorelin compared to a matched placebo flavored oral solution treatment (which contained all the ingredients of the formulation without capromorelin) administered for 4 days. Dogs were randomized into two groups, with Group 1 receiving placebo (0.1 ml/kg) and Group 2 receiving 3.0 mg/kg ENTYCE®. Both groups were treated once a day at approximately 9 AM each day. The first day of dosing was considered Day 0. The placebo and test drug were administered by a syringe placed in the corner of the mouth. The Day 0 weight was used for dose calculations.

### Observations

Dogs were observed at least twice daily and any clinical or behavioral observations were recorded. A physical examination of each dog was completed on Days -14, -2, 0 and on Day 4 at the time of the final body weight measurement. Serum chemistry and hematology (CBC) screening to confirm the health status of each dog prior to enrollment was conducted on Day -2 and repeated on Day 3, approximately 4 to 5 h following administration of the last dose and within 1 h after removal of food, to evaluate any change over the 4 day treatment period. Body weights were recorded prior to feeding on Days -14, -2, 0 and 4.

### Feeding and evaluation of food consumption

Dogs were fed a 25.44% protein nutritionally complete and balanced diet^1^ once daily. The dogs were placed on a time-restricted feeding period beginning 14 days prior to the start of the study. At approximately 10 AM (±30 min) following an overnight fast, dogs were offered 600 grams of food (i.e., twice their normal ration) for a total of 3 h (± 10 min), at which time any remaining food was removed. Food was weighed prior to and after food offering. Food consumption was recorded daily from Day -14 through Day 3.

### Masking

Individuals making any observations on the condition of the dogs, including physical examinations, and any individuals recording data on food consumption and body weight were masked to treatment group.

### Statistical analyses

For food consumption, the baseline value was defined as the mean of the values collected on Day -3, Day -2 and Day -1. The treatment period was the mean of the values collected from Day 0 through Day 3 (4 days) and the percent change was calculated between baseline average and the treatment period average. For body weight, the baseline value was the value obtained at Day 0. Percent changes from baseline were calculated at Day 4, the treatment period for body weight.

Descriptive statistics (number of subjects, mean, standard deviation, standard error of the mean, minimum, median and maximum values) were presented for food consumption percent change at each day measured and body weight percent change, for the treatment period, and baseline. All tests of significance were 2-sided and performed at alpha = 0.05. The SAS® software system,^2^ Version 9.2, was used for all calculations.

Analysis of variance (ANOVA) modeling was utilized to evaluate differences between the treated and placebo groups. The ANOVA model contained terms for treatment, sex and the interaction of treatment by sex. When the interaction term was statistically significant then the treatment group comparisons were carried out for each sex separately. Otherwise, the treatment group comparisons were carried out across the pooled sexes.

In addition, the treatment groups were compared by permutation testing using the SAS® procedure MULTTEST. When, in the ANOVA described above, either the treatment by sex interaction or the sex main effect was significant, the treatment groups were compared separately for each sex. Otherwise, the treatment groups were compared across the pooled sexes. Also, within treatment group, comparisons were assessed by the one-sample *t*-test or Wilcoxon signed rank test, as appropriate, to evaluate the percent change from baseline for body weight and food consumption. The Shapiro-Wilk test for normality was conducted, at the 0.05 significance level, to determine the appropriateness of the one-sample *t*-test. When statistically significant, the Wilcoxon signed rank test was conducted.

## Results

### Clinical observations

The capromorelin and placebo oral solutions appeared to be well tolerated. Salivation was observed repeatedly in all dogs of the capromorelin group post-dosing during all treatment days and in two dogs administered placebo only once on Day 0. Emesis occurred once in two placebo treated dogs during the three days prior to treatment and once in one dog of the capromorelin group during the treatment period. All dogs were considered healthy for the study by the physical examination completed on Day -14. No observations of concern were noted and there were no treatment-related findings in physical examinations performed at the end of the study.

### Serum chemistry and hematology

There were no treatment related changes in serum chemistry or hematology parameters.

### Food consumption and body weight

**Fig. 1:**
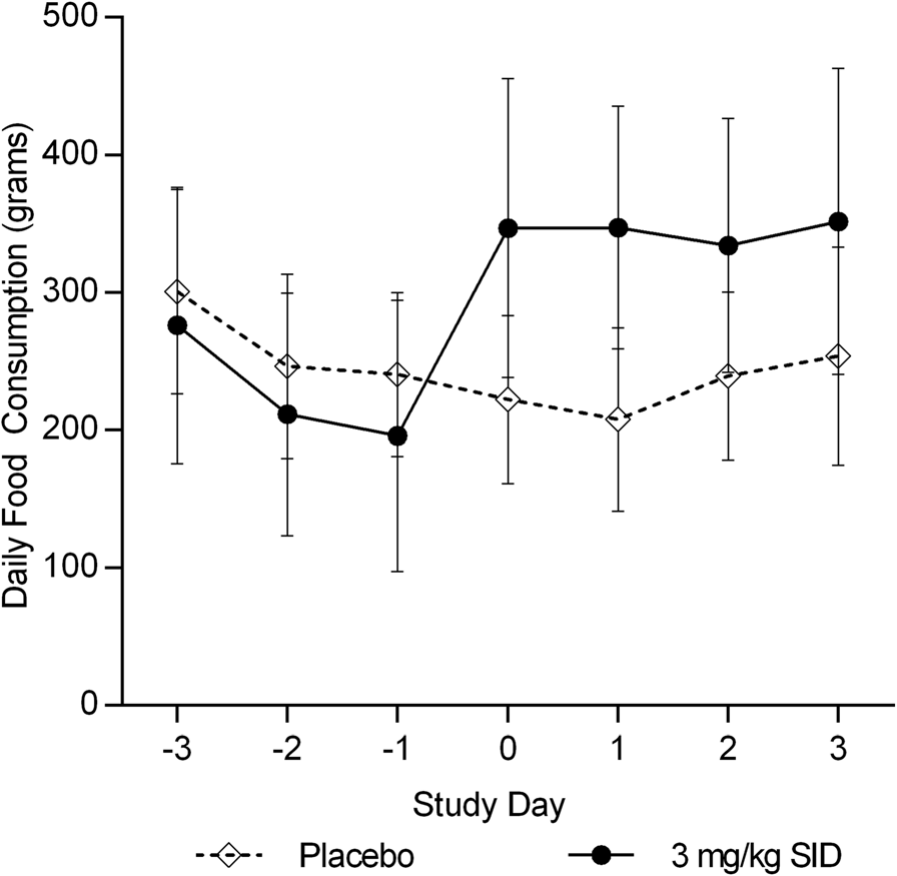
Mean (±SD) food consumption in dogs (*n* = 6 males and 6 females/group) treated with placebo (◊) or capromorelin (●) oral solution. Day 0 is the first day of dosing. Capromorelin resulted in statistically significantly greater mean food consumption as compared to placebo (*P* < 0.001)

There were no statistically significant differences (*P = 0.31*) in results for food consumption or body weight between male and female dogs, so data were pooled. Dogs receiving capromorelin had food consumption that was significantly greater than dogs treated with placebo. Capromorelin treated dogs had increased mean (±SD) food consumption compared to placebo treated dogs (60.55 ± 39.87% versus -11.15 ± 14.23% respectively, *P* < 0.001). Mean food consumption for each group is presented in Fig. 1. The mean percent changes in food consumption in each group are presented in Table 1. All dogs in the capromorelin group gained weight from Day 0 to Day 3. Treated dogs also had increased mean body weights compared to placebo treated dogs (5.96 ± 1.76% versus 0.053 ± 1.14% respectively, *P* < 0.001). The mean percentage changes in body weight for each group are presented in Table 1. The dogs treated with capromorelin had a mean gain of 0.52 kg while the placebo treated dogs had a mean weight gain of 0.004 kg.

**Table 1:** Food consumption and body weight percent changes in dogs treated with either placebo or capromorelin

	Percent food consumption change (Mean ± SD)	Percent weight increase from day 0 to day 3 (Mean ± SD)
Capromorelin	60.55 ± 39.87^a^	5.96 ± 1.76
Placebo	-11.15 ± 14.23^b^	0.053 ± 1.14
*P* value	< 0.001	< 0.001

Dogs (*n* = 6 males, 6 females/group treated for 4 days with either placebo or capromorelin oral solution at 3 mg/kg/day

^a^mean increase of 117.6 grams daily

^b^mean decrease of 30.4 grams daily

## Discussion

Capromorelin oral solution appeared to be well tolerated. The salivation observed in the capromorelin group post-dosing during all treatment days was not considered an adverse event as it is an expected pharmacologic action of capromorelin. Salivation can be caused by a central mechanism in response to the smell or taste of food, which stimulates areas of the brain that influence both the salivary nuclei and appetite center [11]. In humans, the ghrelin receptor has been shown to be present in the salivary glands [12] but this has not yet been demonstrated in dogs. Given that the emesis occurred sporadically in both treatment groups during the acclimation and treatment periods, this finding was considered incidental.

Laboratory dogs were initially utilized to measure the effects of capromorelin oral solution because of the difficulties of measuring food consumption in client-owned dogs on a variety of diets. In laboratory dogs, food consumption and body weight can be carefully measured so that the effect of capromorelin could be demonstrated. Increased food consumption in laboratory dogs confirms data from clinical studies that show an increase in owner assessed appetite in dogs receiving capromorelin oral solution. Therefore, it has been established that dogs treated with capromorelin oral solution have an increased appetite which leads to increased food consumption and increased body weight.

This study showed an increase in food consumption, as expected, in the group receiving the capromorelin 4-day treatment. The large increase in food consumption in the capromorelin treatment group and the resulting increase in body weight over such a short treatment period demonstrates the robust effect of capromorelin. These changes in food consumption and body weight confirm results from an earlier study in laboratory dogs [7]. In that initial laboratory study, the capromorelin effects on food consumption and body weight were demonstrated in a small population of Beagles that included 6 male dogs per group. The current study included 12 dogs, with 6 of each sex per treatment group and had greater statistical power.

A limitation of this study is the short duration; additional studies of longer duration will be needed to demonstrate continued effect of daily treatment. Single breed studies (in Beagles) also need to be confirmed in studies of client-owned dogs.

## Conclusions

In summary, capromorelin has been demonstrated to be effective in increasing food consumption in healthy Beagle dogs and this increased food consumption results in increases in body weight over four days of treatment. The results of this study, along with confirmation that capromorelin increases appetite and body weight in dogs suffering from clinical inappetence, indicate that capromorelin may be a useful new tool for the stimulation of appetite.

## Abbreviations

FDA: Food and Drug Administration
GH: Growth hormone
GHS: Growth hormone secretagogue
GHS-R: Growth hormone secretagogue receptor
IGF-1: Insulin-like growth factor 1
kg: Kilogram
mg: Milligram
SD: Standard deviation

## Footnotes

1 Lab Diet® 25% protein certified canine diet 5007, PMI Nutrition International; St Louis, MO 63144

2 SAS Institute Inc., Cary, NC 27513
